# Manipulation of Band Degeneracy and Lattice Strain for Extraordinary PbTe Thermoelectrics

**DOI:** 10.34133/2020/8151059

**Published:** 2020-01-24

**Authors:** Yixuan Wu, Pengfei Nan, Zhiwei Chen, Zezhu Zeng, Siqi Lin, Xinyue Zhang, Hongliang Dong, Zhiqiang Chen, Hongkai Gu, Wen Li, Yue Chen, Binghui Ge, Yanzhong Pei

**Affiliations:** ^1^Interdisciplinary Materials Research Center, School of Materials Science and Engineering, Tongji Univ., 4800 Caoan Rd., Shanghai 201804, China; ^2^Institute of Physical Science and Information Technology, Anhui University, Hefei 230601, China; ^3^Department of Mechanical Engineering, The University of Hong Kong, Pokfulam Road, Hong Kong SAR, China; ^4^Center for High Pressure Science and Technology Advanced Research, Shanghai 201203, China; ^5^State Key Laboratory of Superhard Materials, Jilin University, Changchun 130012, China

## Abstract

Maximizing band degeneracy and minimizing phonon relaxation time are proven to be successful for advancing thermoelectrics. Alloying with monotellurides has been known to be an effective approach for converging the valence bands of PbTe for electronic improvements, while the lattice thermal conductivity of the materials remains available room for being further reduced. It is recently revealed that the broadening of phonon dispersion measures the strength of phonon scattering, and lattice dislocations are particularly effective sources for such broadening through lattice strain fluctuations. In this work, a fine control of MnTe and EuTe alloying enables a significant increase in density of electron states near the valence band edge of PbTe due to involvement of multiple transporting bands, while the creation of dense in-grain dislocations leads to an effective broadening in phonon dispersion for reduced phonon lifetime due to the large strain fluctuations of dislocations as confirmed by synchrotron X-ray diffraction. The synergy of both electronic and thermal improvements successfully leads the average thermoelectric figure of merit to be higher than that ever reported for p-type PbTe at working temperatures.

## 1. Introduction

Driven by energy crisis and the resultant environmental issues, interests in advancing clean energy technologies have grown rapidly in this century. Based on either Seebeck or Peltier effect, thermoelectrics have important applications in energy harvesting, refrigeration, and thermal sensing, due to the capability of a direct conversion between heat and electricity [1–3]. To enable a widespread application, the conversion efficiency needs to be maximized, which requires thermoelectric materials to have a high figure of merit *zT* = *S*^2^*σT*/(*κ*_*E*_ + *κ*_*L*_). In details, *S*, *σ*, *T*, *κ*_*E*_, and *κ*_*L*_ are the Seebeck coefficient, electrical conductivity, absolute temperature, and electronic and lattice components of the thermal conductivity, respectively [4].

Ideally, *zT* can be maximized when both electronic transport properties and lattice thermal conductivity are fully optimized [5–7]. However, critical electronic parameters determining *zT*, including *S*, *σ*, and *κ*_*E*_, are strongly intercorrelated, leading to the difficulty of improving *zT* through an individual manipulation of a certain parameter. A great deal of efforts have been devoted to decoupling these parameters for an effective enhancement in power factor *S*^2^*σ*, and successful strategies are typified by engineering the band structure for a high band degeneracy [8–11], a low electron inertial mass [12], and a weak charge scattering [13], which has been demonstrated in various thermoelectrics [4, 14–19].

Alternatively, a minimization in lattice thermal conductivity (*κ*_*L*_) has led to great *zT* enhancements as well, which can be realized either by strengthening the scattering of phonons [20–23] in known materials or by exploring new materials with a intrinsically low *κ*_*L*_ [24]. For the former aspect of phonon scattering, it is recently revealed that the essence can be attributed to the broadening of phonon dispersion. The ideal phonon dispersion (frequency versus wave vector) is a curve called the “ground state” at 0 K. In fact, due to the existence of anharmonic lattice vibrations at finite temperatures in real materials, the resultant dynamic interatomic-force-constant fluctuations would lead the phonon dispersion to show frequency fluctuations around the “ground state,” which equivalently leads to a widening of the phonon dispersion “curve.” In addition to inherent lattice anharmonicity, various defects [4, 25] could induce fluctuations in atomic mass and/or in interaction force constant for a further broadening [26–29]. For the latter case of intrinsically low *κ*_*L*_, demonstrated strategies include a strong lattice anharmonicity [30, 31], a low sound velocity [32] in loosely bonded materials with heavy and diffusive species [33, 34], and a low fraction of acoustic phonons [35] enabled in a complex crystal structure [36–38].

These approaches have been frequently proven to be successful for advancing thermoelectrics, especially in p-type IV-VI compounds [16, 39–47]. Manipulation of the energy offset between the light valence band (*L*) and the heavy valence band (*Σ*) via alloying enables charge carriers to be transported by many band valleys (band degeneracy) for an increase in *σ* without explicitly reducing *S* [48]. With years of exploration, effective alloying species for converging the valence bands of conventional PbTe thermoelectrics at desired temperatures are found to be monotellurides including MnTe [49], MgTe [50], CdTe [51], YbTe [52], EuTe [53], SrTe [54], and SnTe [55]. These alloys have shown greatly enhanced *zT*, yet the lattice thermal conductivity seems not to be all simultaneously minimized by the alloying process [49, 51, 56].

It is therefore motivated that a synergic approach on minimizing *κ*_*L*_ by various defects for a further enhancement in *zT* [57–62]. Since all these defects would introduce fluctuations in atomic mass and/or in lattice strain [27, 28, 63, 64], both of which would lead to fluctuations in phonon frequency at a given wave vector (i.e. broadening in phonon dispersion) [26]. This accelerates phonons to relax back to its equilibrium states (from the “excited states” to the widened “ground states”) since available frequencies are much more diversified in a broadened phonon dispersion [65]. Efforts have been often devoted on 0D point defects [28, 66–68] and 2D boundary interfaces [2, 69–74] for minimizing *κ*_*L*_, which are, respectively, considered to be effective sources for scattering phonons with high and low frequencies [25, 75, 76]. Recently developed technique for introducing in-grain dislocations in IV-VI thermoelectrics is particularly interesting, not only because the resultant 1D defects are effective for scattering phonons with midrange frequencies [22, 77–80] but also dislocations could enable a reduction in *κ*_*L*_ nearly at no expense of a reduction in carrier mobility [26].

In order to maximize the thermoelectric performance of p-type PbTe, this work focuses on a full optimization of valence band convergence by alloying with EuTe and MnTe. Simultaneously, Na-doping is used to ensure not only a sufficiently high dislocation density for a maximization in lattice strain fluctuations but also a high enough carrier concentration. The well-improved electronic performance enabled by converged valence bands and the significantly reduced *κ*_*L*_ due to dislocations induced lattice strain fluctuations, synergistically lead to an extraordinary thermoelectric performance.

## 2. Results and Discussions

The details on synthesis, characterization, transport property measurements, and band structure calculations are given in the Supporting Information. X-ray diffraction (including synchrotron X-ray) was used to characterize the phase compositions and to estimate the lattice parameters. As shown in [Supplementary-material supplementary-material-1], observable diffraction peaks can be well indexed to the rock salt structure of PbTe. The lattice parameter decreases linearly with the increase of MnTe concentration, which can be understood by the smaller size of Mn as compared to that of Pb. The carrier concentration of Na_*y*_Eu_0.03_Mn_0.03_Pb_0.94-__*y*_Te saturates at *y* > 3% ([Supplementary-material supplementary-material-1]), indicating a Na solubility at this nominal concentration.

### 2.1. Electronic Transport Properties

PbTe alloying with EuTe and MnTe have been proven to effectively converge the valence bands and increase the band gap of PbTe [49, 53]. Previous work further indicated that the thermoelectric performance optimized at a concentration of ~3% EuTe alloying [53] or a concentration of ~4% MnTe alloying [49]. Importantly, an increase of Na-doping in PbTe-3%EuTe alloys is found to enable a controllable transition of dominant types of defect form 0D substitutions to 1D in-grain dislocations and then to 2D interfaces induced by nanoprecipitation [53]. According to these results, this work focuses on PbTe-3%EuTe alloys as the parent material with further MnTe alloying and Na doping for optimizing both the band structure, carrier concentration, and microstructure.

**Figure 1:**
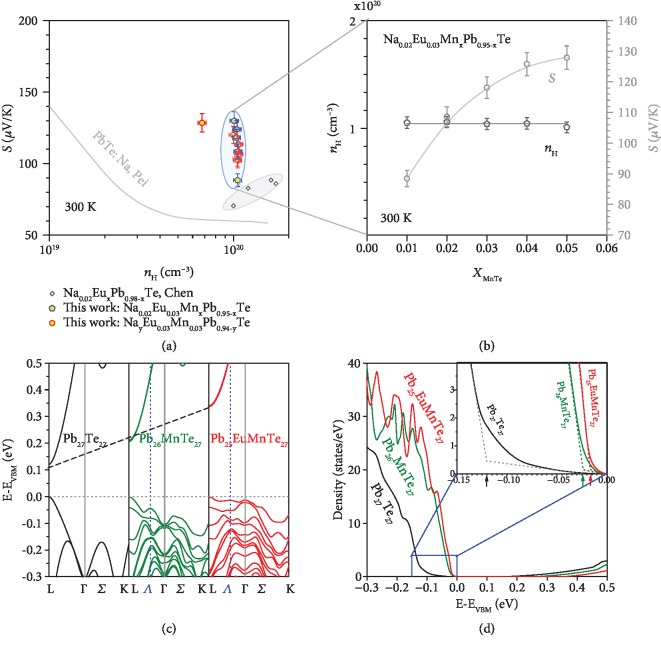
Room temperature Seebeck coefficient (*S*) versus Hall carrier concentration (*n*_*H*_) for various series of PbTe alloys from this work and literatures [53, 56] (a) and MnTe-alloying concentration-dependent Seebeck coefficient and Hall carrier concentration at room temperature (b) for Na_0.02_Eu_0.03_Mn_*x*_Pb_0.95-__*x*_Te synthesized in this work. Calculated band structures (c) and density of states (d) of Pb_27_Te_27_, Pb_26_MnTe_27_ and Pb_25_EuMnTe_27_ with a setting of valence band maximum (VBM) at 0 eV.

Figure 1(a) shows the room temperature carrier concentration-dependent Seebeck coefficient for various series of PbTe alloys from this work and literature [53, 56]. It is shown that further alloying with MnTe in this work indeed leads to a further increase in the Seebeck coefficient. In more details, an increase of MnTe-alloying concentration does not lead to an observable change in carrier concentration when *a* > 2% Na doping is used, but the Seebeck coefficient continuously increases (Figure 1(b)).

This could be understood by the MnTe-alloying which induced further optimization in the valence band structure, as evidenced from the band structure calculations shown in Figures 1(c) and 1(d). The details on density functional theory (DFT) calculations are given in the supplementary. It is seen that the valence band maximum (VBM) and the conduction band minimum (CBM) locate at *L* point for Pb_27_Te_27_, Pb_26_MnTe_27_ and Pb_25_EuMnTe_27_. In addition, the direct band gap at *L* increases due to MnTe and EuTe alloying [53]. Furthermore, MnTe and EuTe alloying [53] effectively reduces the energy offsets between the valence bands, leading to an involvement near the VBM of many bands for charge transport. Therefore, a superior electronic performance can be expected in these PbTe-EuTe-MnTe alloys. It should be noted that a supercell is needed to simulate the doping systems, and a back-folding technique is used for all the materials including pristine PbTe to enable a self-consistent comparison on DOS change due to doping. Similarly, an involvement of multiple transporting valence bands has been observed as well in SnTe heavily alloyed with MgTe [81].

**Figure 2:**
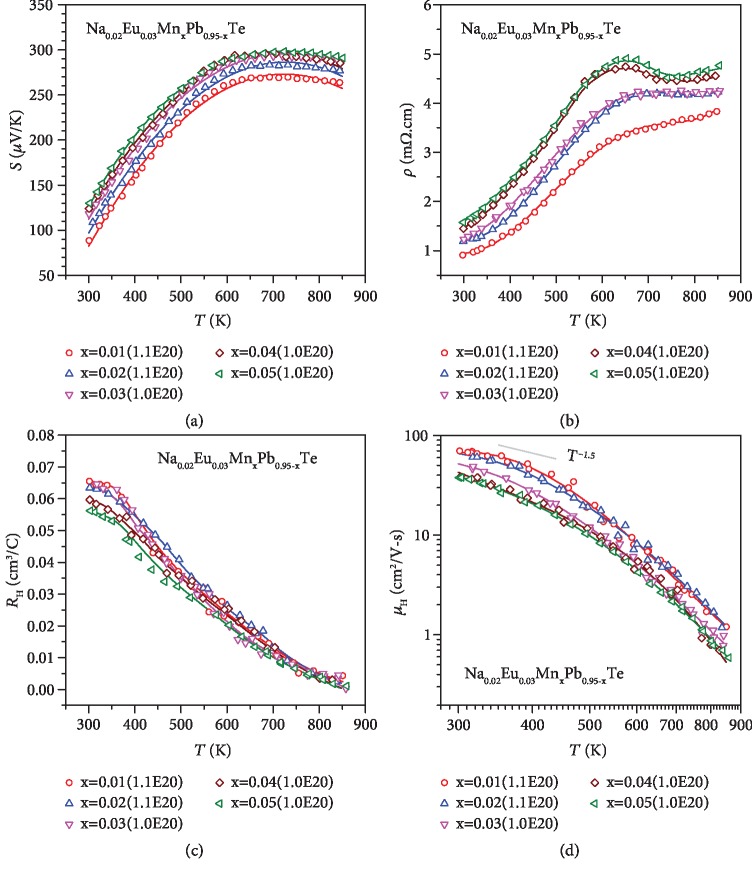
Temperature-dependent Seebeck coefficient (a), resistivity (b), Hall coefficient (c), and Hall mobility (d) for Na_0.02_Eu_0.03_Mn_*x*_Pb_0.95-__*x*_Te.

The density of states (DOS) for Pb_27_Te_27_, Pb_26_MnTe_27_, and Pb_25_EuMnTe_27_ are shown in Figure 1(d). It is seen that the DOS near the valence band edge increases with MnTe and EuTe alloying. This indicates that alloying with EuTe and MnTe are indeed helpful for converging possibly transporting valence bands for maximizing the Seebeck coefficient. The increase in density of state due to MnTe and EuTe alloying can further be confirmed by optical measurements ([Supplementary-material supplementary-material-1] and [Supplementary-material supplementary-material-1]), where the inertial effective mass determined by the Lyden method [82] shows a similar increase with increasing MnTe alloying at a given carrier concentration of ~1.05 × 10^20^ cm^−3^. This helps understand the reduction in mobility observed (Figure 2(d)), in addition to charge scattering due to defects. The 3% MnTe and EuTe alloying are focused in this work, because these contents of 3% are the optimized ones to achieve the highest electronic properties in PbTe-MnTe and PbTe-EuTe solid solutions, according to previous works [49, 53].

**Figure 3:**
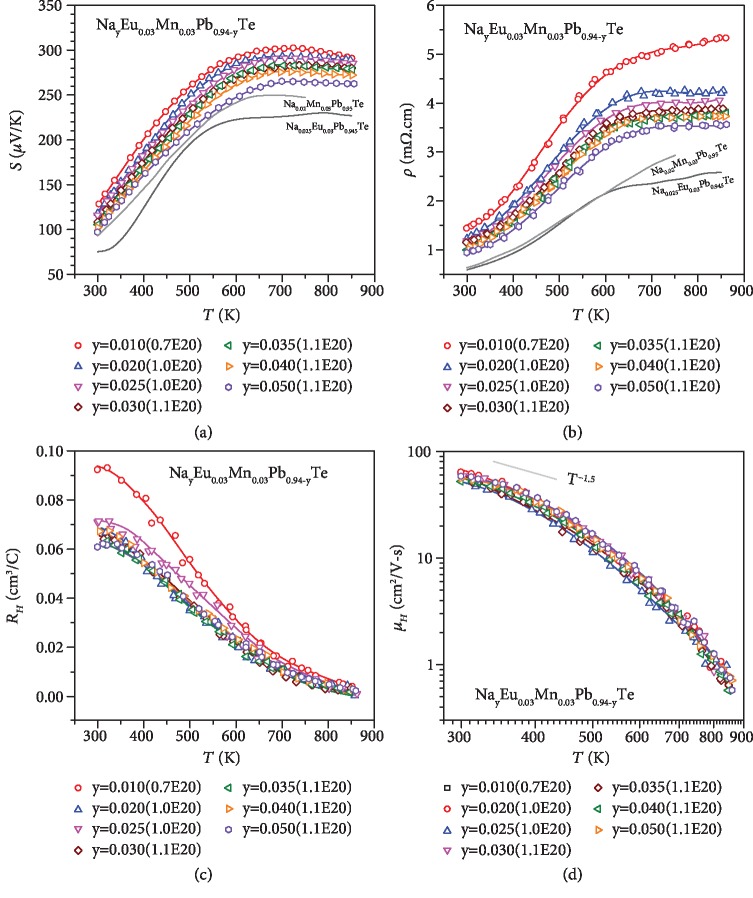
Temperature-dependent Seebeck coefficient (a), resistivity (b), Hall coefficient (c), and Hall mobility (d) for Na_*y*_Eu_0.03_Mn_0.03_Pb_0.94-__*y*_Te, with a comparison to literature results [49, 53].

The detailed temperature-dependent electronic transport properties including the Seebeck coefficient, resistivity, Hall coefficient, and Hall mobility for Na_0.02_Mn_*x*_Pb_0.98-__*x*_Te (*x* ≤ 0.05) and Na_*y*_Eu_0.03_Mn_0.03_Pb_0.94-__*y*_Te (*y* ≤ 0.05) are shown in Figures 2 and 3, respectively. In pristine p-type PbTe, the Hall coefficient peaks at about 450 K [83], which can be a rough indication of valence band convergence. In this work, such a peaking in Hall coefficient is not observable, which corresponds to well-converged valence bands in the entire temperature range. Temperature-dependent Hall mobility all show an unchanged dominant carrier scattering by acoustic phonons.

### 2.2. Thermal Transport Properties

A full enhancement in performance relies largely on a minimization in lattice thermal conductivity (*κ*_*L*_). Phonon transport is essentially determined by the phonon dispersion and its broadening. In addition to inherent phonon scattering due to lattice anharmonicity, various defects could create mass and strain fluctuations [28, 64] for broadening phonon dispersion [27]. Such broadening leads to a shortening in phonon relaxation time for a reduced *κ*_*L*_. Without an explicit variation in composition, as the case here that a ~3%MnTe+3%EuTe alloying is focused on because of its capability for well-converged valence bands, manipulation of lattice strain fluctuation becomes an important avenue for reducing *κ*_*L*_ further.

**Figure 4:**
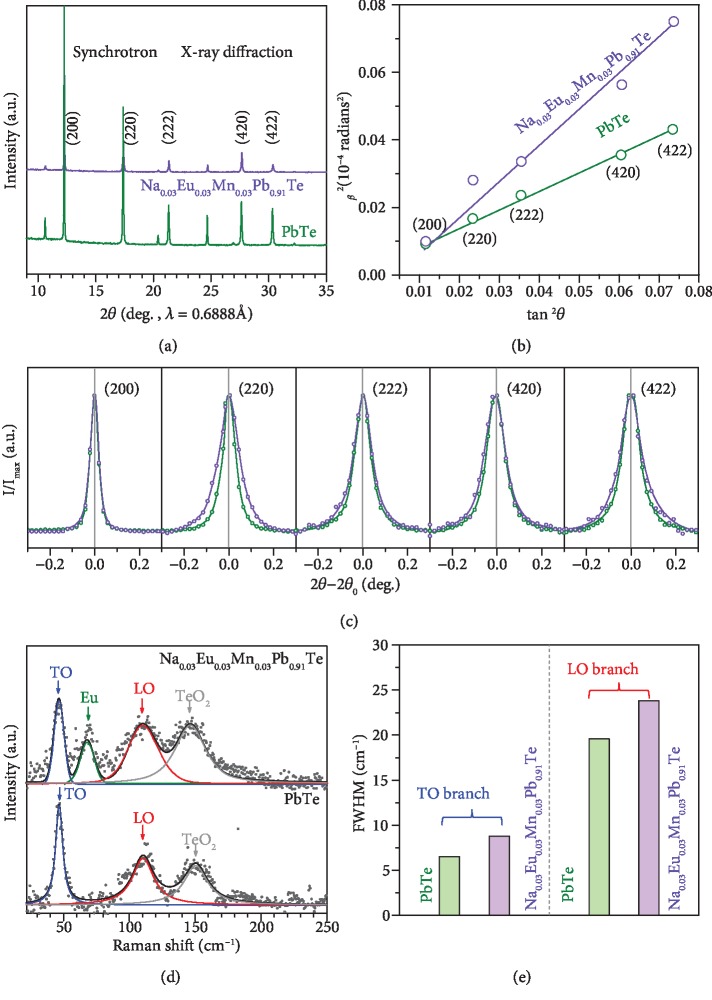
Synchrotron X-ray diffraction patterns (a), strain analysis (b), detailed broadening of diffraction peaks (c), Raman spectrum with Lorentzian deconvolutions (d), and the resulting Raman peak broadening (e) for pristine PbTe and Na_0.03_Eu_0.03_Mn_0.03_Pb_0.91_Te at room temperature.

Theoretically, lattice strain fluctuations would cause the broadening of phonon dispersion due to the local expansion and compression. Therefore, X-ray diffraction and Raman scattering techniques are the macroscopic, convenient, and reliable methods to determine the lattice strains and phonon dispersion broadening. Once lattice strain fluctuations exist, a broadening and a reduction in intensity of XRD peaks will be observed [84]. The details on lattice strain estimation from X-ray diffraction are given in the supplementary. [Supplementary-material supplementary-material-1] shows the synchrotron X-ray diffraction patterns, Figure 4(b) shows the corresponding strain analyses for PbTe and Na_0.03_Eu_0.03_Mn_0.03_Pb_0.91_Te with detailed broadening of diffraction peaks (Figure 4(c)). *β* in Figure 4(b) is the “full width at half maximum” of the intense diffraction peaks and *θ* is the Bragg angle. A much larger slope for Na_0.03_Eu_0.03_Mn_0.03_Pb_0.91_Te corresponds to the larger lattice stain fluctuations induced by dislocation as will be discussed below.

Furthermore, lattice strain fluctuations could result in local symmetry breaking of the ideal rock-salt structure of PbTe, which further leads the optical modes at Brillouin zone center including pristine PbTe to be first-order Raman active [85]. The broadening in Raman peaks of both transverse optical (TO) and longitudinal optical (LO) modes at Γ point enables a direct indication of the broadening in phonon dispersion due to lattice strain fluctuations [86]. To estimate the positions of active modes induced by impurities (Eu, Mn, and Na), an extended linear diatomic-chain model is utilized for estimating the frequencies of the local modes [87]. As shown in Figure 4, Raman peaks at about 47 cm^−1^, 74 cm^−1^, 108 cm^−1^, and 150 cm^−1^, respectively, correspond to the TO modes, Eu impurity, LO modes, and TeO_2_ [86, 88]. The solid curves show the deconvolution using a Lorentzian approximation. It should be noted that it is difficult to distinguish the peaks of Mn^2+^ at ~99 cm^−1^ and Na^+^ at ~142 cm^−1^ because of the possible overlapping with those of LO at 108 cm^−1^ and TeO_2_ at ~148 cm^−1^. Similar to literature Raman results of PbTe materials [89, 90], broadening of Raman peak of TO and LO modes can be observed in this work, which confirms the existence of lattice strains and the strain-induced force constant fluctuations leading to a broadening in the phonon dispersion. Similar Raman peak broadening due to existence of lattice strains are frequently observed in many semiconductors [91–93].

**Figure 5:**
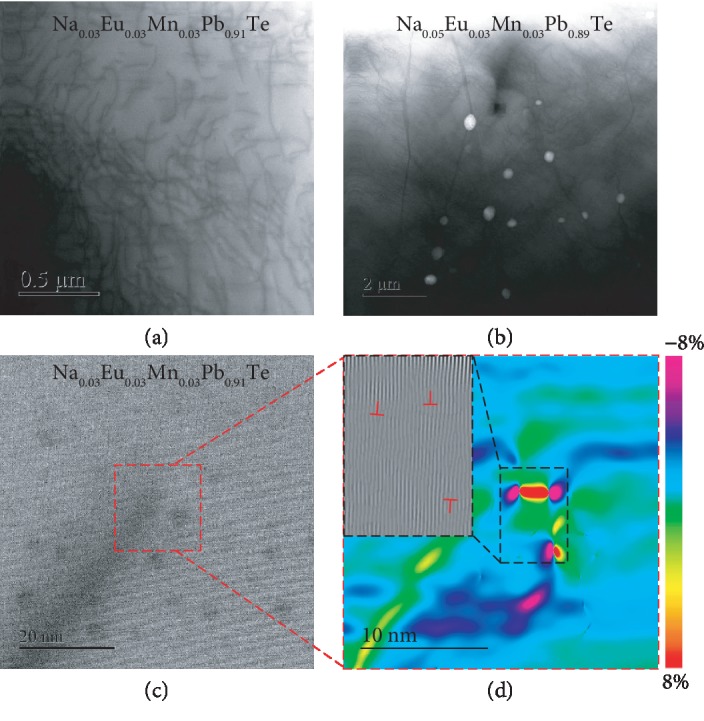
Low-magnification STEM images for Na_0.03_Eu_0.03_Mn_0.03_Pb_0.91_Te (a) and Na_0.05_Eu_0.03_Mn_0.03_Pb_0.89_Te (b), showing dense dislocations and a few nanoprecipitates (white spots). High-magnification STEM image (c) and the corresponding strain mappings (d) for Na_0.03_Eu_0.03_Mn_0.03_Pb_0.91_Te. Both samples are annealed at 900 K for 48 hours to reach the dislocation equilibrium.

**Figure 6:**
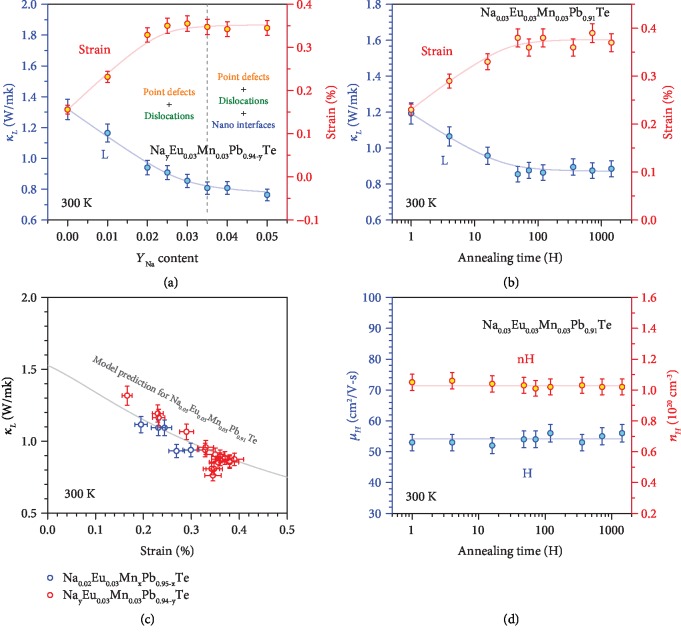
Composition-dependent lattice strains and lattice thermal conductivity (*κ*_*L*_) for Na_*y*_Eu_0.03_Mn_0.03_Pb_0.94-__*y*_Te with a 48-hour annealing at 900 K (a); annealing time-dependent lattice strain (at 900 K), and *κ*_*L*_ for Na_0.03_Eu_0.03_Mn_0.03_Pb_0.91_Te (b); lattice strain-dependent *κ*_*L*_ with a comparison to model prediction for Na_0.03_Eu_0.03_Mn_0.03_Pb_0.92_Te (c); and annealing time-dependent Hall mobility and carrier concentration (d) for Na_0.03_Eu_0.03_Mn_0.03_Pb_0.91_Te.

Microscopically, scanning transmission electron microscopy (STEM) is used to reveal the origin of lattice strains. As shown in Figure 5, very dense in-grain dislocations are observable in Na_0.03_Eu_0.03_Mn_0.03_Pb_0.91_Te. To analyze the lattice strain induced by dislocations, typical high-magnification STEM images are focused. Based on a geometric phase analysis (GPA), which is a semiquantitative lattice image-processing approach, spatially distributed strain fields can be mapped (Figure 5(d)). It can be seen that both tensile and compressive strains concentrated around the dislocations are as large as a few percentages, which enables a large average strain fluctuation of ~0.4% in the entire material of Na_0.03_Eu_0.03_Mn_0.03_Pb_0.91_Te according to the X-ray diffraction (Figure 6(a)). As shown in Figures 5, [Supplementary-material supplementary-material-1], it is interesting that a further increase of Na-doping concentration leads to a coexistence of both dense dislocations and Pb-poor nanoprecipitates, where the heterogeneous precipitates are confirmed by electron diffraction and energy dispersion spectrum (EDS) analyses ([Supplementary-material supplementary-material-1]). Similar to literature results, the average lattice strains saturate at *y* ≥ 3% in Na_*y*_Eu_0.03_Mn_0.03_Pb_0.94-__*y*_Te alloys (Figure 6(a)), indicating that nanoprecipitates do not introduce significant lattice strain fluctuations [80].

A direct observation of dislocation formation and movement is generally challenging, and it is believed that clustering of vacancies is important for these processes. Na doping in this work leads to a high hole concentration of ~10^20^ cm^−3^, which could promote the formation of oppositely charged anion vacancies due to charge compensation. Driven by thermodynamics and dynamics such as annealing at high temperatures, clustering/collapsing of vacancies and dislocation climb could lead to the formation and multiplication for dense dislocations at equilibrium [22, 53]. A control experiment in Na_0.03_Eu_0.03_Mn_0.03_Pb_0.91_Te with different annealing durations at 900 K enables an observation of dislocation equilibrium taking a few dozen hours, as indicated by the saturation in lattice strain in long-term (≥48 hours) annealed samples (Figure 6(b)). Interestingly, these in-grain dislocations are found to be sable up to at least 2 months at the annealing temperature of 900 K, and the underlying mechanism deserves a further study.

Most importantly, both the increase and saturation in lattice strain for Na_0.03_Eu_0.03_Mn_0.03_Pb_0.91_Te consistently lead to the decrease and saturation in lattice thermal conductivity (Figures 6(a) and 6(b)). Lattice thermal conductivity (*κ*_*L*_) is estimated through subtracting the electronic contribution according to Wiedemann–Franz law (*κ*_*E*_ = *LT*/*ρ*) from total thermal conductivity, where *L* is the Lorenz factor determined via a single parabolic band (SPB) model with acoustic scattering (Figures 2(d) and 3(d)). With a nearly unchanged sound velocity due to alloying and doping ([Supplementary-material supplementary-material-1]), the increase in lattice strains is found to indeed take the major responsibility for the decrease in *κ*_*L*_, which can further be predicted by *κ*_*L*_ modeling without any fitting parameters (Figure 6(c)). Details on *κ*_*L*_ modeling are given in the supplementary ([Supplementary-material supplementary-material-1]). *κ*_*L*_ as low as 0.8 W/m-K at room temperature is one of the lowest ever reported for p-PbTe.

Interestingly, the carrier mobility, carrier concentration, and Seebeck coefficient are found to be nearly unchanged with the variation of lattice strain fluctuations for Na_0.03_Eu_0.03_Mn_0.03_Pb_0.91_Te (Figure 6(d)), although *κ*_*L*_ decreases significantly. The well-maintained mobility in materials with strains can be understood by the high dielectric constant of PbTe and the relatively high temperature range involved in this work, both of which significantly weaken the charge scattering by electrostatic fields of dislocations [26]. Such a nearly independent *κ*_*L*_ reduction induced by strain engineering would enable an effective approach for enhancing PbTe thermoelectrics.

**Figure 7:**
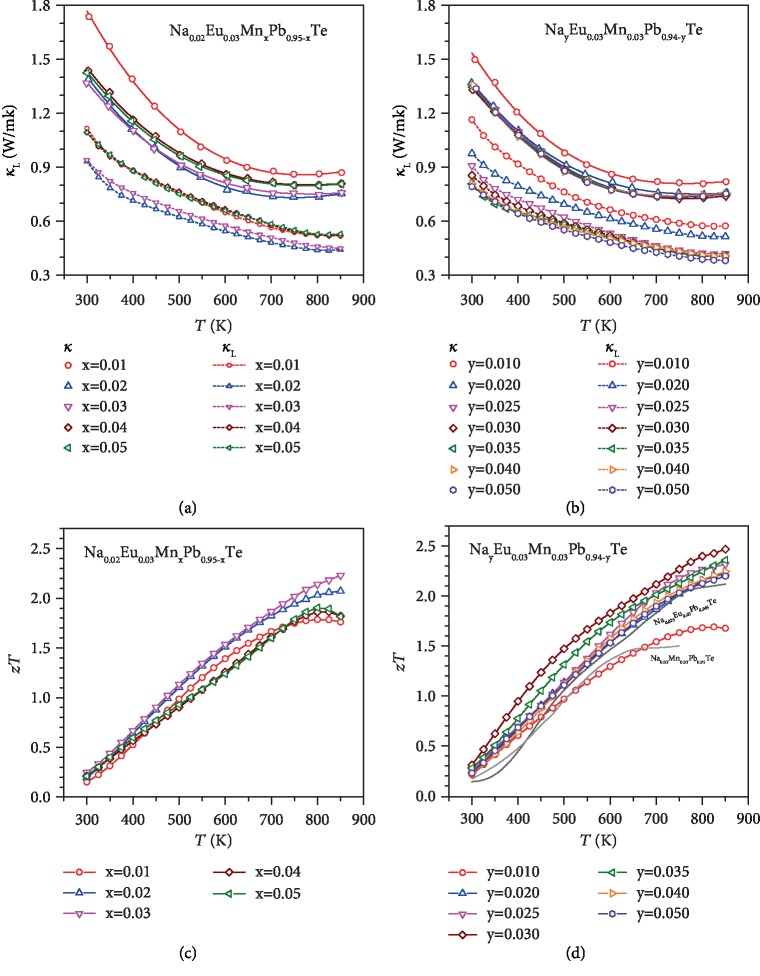
Temperature-dependent total and lattice thermal conductivity (a, b) and thermoelectric figure of merit (c, d) for Na_0.02_Eu_0.03_Mn_*x*_Pb_0.95-__*x*_Te (a, c) and Na_*y*_Eu_0.03_Mn_0.03_Pb_0.94-__*y*_Te (b, d).

**Figure 8:**
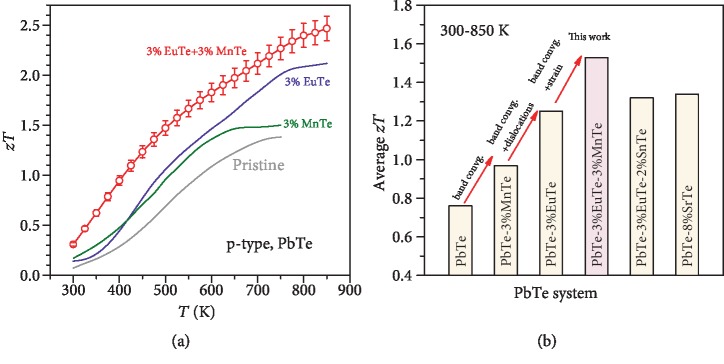
Temperature-dependent thermoelectric figure of merit (with the standard deviation of 12 measurements) and its average within 300-850 K in optimally doped PbTe alloys, with a comparison to literature results [26, 49, 53, 54, 56].

Synergy of superior electronic performance guaranteed by the well-converged valence bands and minimal lattice thermal conductivity (Figures 7(a) and 7(b)) enabled by dislocation-induced lattice strains, successfully leads to a realization of extraordinary *zT* (Figures 7(c) and 7(d)). It should be noted that the slight increase in *κ*_*L*_ at *T* > 750 is due to the bipolar effect as seen from Figure 3.  Figure 8 shows the evolution of *zT* enhancements due to the beneficial effects of band convergence and dislocation-induced lattice strains. It is interesting to note that the average *zT* within 300-850 K of ~1.5 realized in this work is actually higher than that ever reported for p-type PbTe thermoelectrics. Furthermore, the high *zT* is found to be highly reproducible ([Supplementary-material supplementary-material-1]).

## 3. Summary

This work demonstrates the effectivity of MnTe and EuTe coalloying for maximizing the valence band degeneracy as well as of dislocation-induced lattice strains for minimizing phonon relaxation time of PbTe. The resultant synergy of both electronic and thermal approaches successfully leads to a breakthrough in thermoelectric efficiency. It is shown that dislocation-induced lattice strains enable a nearly independent reduction in lattice thermal conductivity, which could in principle open new possibilities for advancing PbTe and similar thermoelectrics.

## Supplementary Materials

Supplementary Materials Materials and Methods: Table S1: parameters used for the modeling. Figure S1: XRD patterns (a-c) and lattice parameters (d-e) for Na_0.02_Eu_0.03_Mn*_x_*Pb_0.95-*x*_Te (a, d), Na*_y_*Eu_0.03_Mn_0.03_Pb_0.94-*y*_Te (b, e), and Na_0.03_Eu_0.03_Mn_0.03_Pb_0.91_Te (c, f) with different annealing time. Figure S2: normalized optical absorption coefficient for Na_0.02_Eu_0.03_Mn*_x_*Pb_0.95-*x*_Te (a) and Na*_y_*Eu_0.03_Mn_0.03_Pb_0.94-*y*_Te (b) at room temperature. Table S2: room temperature Hall carrier concentration (nH), inertial effective mass (mI^∗^), plasma energy, and density (g/cm^3^) for Na_0.02_Eu_0.03_Mn*_x_*Pb_0.95-*x*_Te, and Na*_y_*Eu_0.03_Mn_0.03_Pb_0.94-*y*_Te. Figure S3: TEM images and EDS composition mappings for Na_0.03_Eu_0.03_Mn_0.03_Pb_0.91_Te. Figure S4: low-magnification STEM images (a) and high-magnification STEM image(b), electron diffraction patterns(c), and EDS composition mappings (d-i) for Na_0.05_Eu_0.03_Mn_0.03_Pb_0.89_Te, indicating the coexistence of both dense dislocations and nanoprecipitates. Figure S5: MnTe (a) and NaTe (b) content-dependent sound velocity for Na_0.02_Eu_0.03_Mn*_x_*Pb_0.95-*x*_Te and Na*_y_*Eu_0.03_Mn_0.03_Pb_0.94-*y*_Te at room temperature. Figure S6: repeated measurements for the high-*zT* composition Na_0.03_Eu_0.03_Mn_0.03_Pb_0.91_Te showing highly reproducible and stable thermoelectric properties.
