# Supplementary material for: Manipulation of Band Degeneracy and Lattice Strain for Extraordinary PbTe Thermoelectrics
Source: Research (Wash D C). 2020 Jan 24;2020:8151059. doi: 10.34133/2020/8151059 (PMC7000992; doi:10.34133/2020/8151059)
Supplement: Supplementary Materials — Materials and Methods: Table S1: parameters used for the modeling. Figure S1: XRD patterns (a-c) and lattice parameters (d-e) for Na0.02Eu0.03MnxPb0.95-xTe (a, d), NayEu0.03Mn0.03Pb0.94-yTe (b, e), and Na0.03Eu0.03Mn0.03Pb0.91Te (c, f) with different annealing time. Figure S2: normalized optical absorption coefficient for Na0.02Eu0.03MnxPb0.95-xTe (a) and NayEu0.03Mn0.03Pb0.94-yTe (b) at room temperature. Table S2: room temperature Hall carrier concentration (nH), inertial effective mass (mI∗), plasma energy, and density (g/cm3) for Na0.02Eu0.03MnxPb0.95-xTe, and NayEu0.03Mn0.03Pb0.94-yTe. Figure S3: TEM images and EDS composition mappings for Na0.03Eu0.03Mn0.03Pb0.91Te. Figure S4: low-magnification STEM images (a) and high-magnification STEM image(b), electron diffraction patterns(c), and EDS composition mappings (d-i) for Na0.05Eu0.03Mn0.03Pb0.89Te, indicating the coexistence of both dense dislocations and nanoprecipitates. Figure S5: MnTe (a) and NaTe (b) content-dependent sound velocity for Na0.02Eu0.03MnxPb0.95-xTe and NayEu0.03Mn0.03Pb0.94-yTe at room temperature. Figure S6: repeated measurements for the high-zT composition Na0.03Eu0.03Mn0.03Pb0.91Te showing highly reproducible and stable thermoelectric properties. [file 8151059.f1.pdf]

Supplementary

Manipulation of Band Degeneracy and Lattice Strain for Extraordinary PbTe Thermoelectrics

Yixuan Wu[1], Pengfei Nan[2], Zhiwei Chen[1], Zezhu Zeng[3], Siqi Lin[1], Xinyue Zhang[1], Hongliang Dong[4], Zhiqiang Chen[4], Hongkai Gu[4,5], Wen Li[1], Yue Chen[3], Binghui Ge[2,*] and Yanzhong Pei[1,*]

[1]Interdisciplinary Materials Research Center, School of Materials Science and Engineering, Tongji Univ., 4800 Caoan Rd., Shanghai 201804, China.

[2]Institute of Physical Science and Information Technology, Anhui University, Hefei 230601, China

[3]Department of Mechanical Engineering, The University of Hong Kong, Pokfulam Road, Hong Kong SAR, China

[4] Center for High Pressure Science and Technology Advanced Research, Shanghai 201203, China

[5]State Key Laboratory of Superhard Materials, Jilin University, Changchun 130012, China

[*]Email: bhge@ahu.edu.cn (BG); yanzhong@tongji.edu.cn (YP)

Materials and Methods:

Polycrystalline $Na_{0.02}Eu_{0.03}Mn_xPb_{0.95-x}Te$ (x≤0.05) and $Na_yEu_{0.03}Mn_{0.03}Pb_{0.94-y}Te$ (y≤0.05) were synthesized by melting the stoichiometric amount of high purity elements (>99.99%) at 1300 K for 3 hours, quenching in cold water followed by a further annealing at 900 K for up to 2 months. MnTe- and EuTe-alloying were used to tune the band structure. Na-doping was used to optimize the carrier concentration and to control the microstructure. The annealed ingots were hand ground into powders for identifying the phase composition and density (all >98% of the theoretical density). The powders were densified by hot pressing under a uniaxial pressure of ~60 MPa at 877 K for 30 minutes. The obtained pellets were ~12 mm in diameter and ~1.6 mm in thickness for property measurements.

The phase composition of the samples was identified by X-ray diffraction (Synchrotron XRD and DX2700). Synchrotron XRD experiments are conducted in reflection mode at the BL14B1 beamline of the Shanghai Synchrotron Radiation Facility (SSRF). The energy of the monochromatic X-ray beam is 18 keV, corresponding to a wavelength of 0.6888 Å. The Raman spectra were excited by the 532 nm lines of an argon laser in the back-scattering geometry, using a Jobin Yvon model U-1000 monochromator equipped with a conventional photo-counting system. The sintered samples (pellet in geometry) were held onto a glass slide for Raman measurements. In order to ensure a better signal-to-noise ratio, 4 sets of measurements were counted for averaging.

The electronic transport properties including Seebeck coefficient (S), Hall coefficient (R_H), and resistivity ($\rho=1/\sigma$) of the pellet samples were measured from 300 to 850 K. The Seebeck coefficient was obtained from the slope of the voltage vs. temperature gradients of 0-5 K; the resistivity and Hall coefficient were measured using van der Pauw technique with a reversible magnetic field of 1.5 T. The measurement uncertainty for S, ρ and κ is 5% approximately. The thermal diffusivity (λ) was measured by a laser flash technique (Netzsch LFA457). The thermal conductivity (κ) was determined by $\kappa = \lambda C_p d$; where d was the density estimated by a mass/volume method and C_p was determined from the measurements of Blachnik by $C_p(k_B/\text{atom})=3.07+0.00047(T/\text{K}-300)$ for lead chalcogenides[1, 2]. Optical reflectance was measured by a Fourier Transform Infrared Spectroscopy (FTIR, Bruker Tensor II equipped with a diffuse reflectance attachment) at room temperature.

The microstructure was characterized by Bright Field (BF) and High-Angle Annular Dark Field (HAADF) imaging in Scanning Transmission Electron Microscopy (STEM) mode. STEM specimens were prepared by mechanical slicing, polishing, and dimpling, and followed by ion-milling with liquid nitrogen.

To study the effects of alloying on the band structure and density of states of PbTe, a 3×3×3 supercell of the rock-salt primitive cell was used. Density functional theory (DFT) calculations were performed with VASP [3, 4] using Perdew-Burke-Ernzerhof functional (PBE) of projector augmented wave (PAW) method[5]. For $Pb_{26}MnTe_{27}$, one Pb atom in the supercell was substituted by the alloying atom arbitrarily, whereas quasi-random structures were generated for $Pb_{25}EuXTe_{27}$ using USPEX[6]. An energy cutoff of 400 eV was applied. We used the Γ-centered Monkhorst-Pack k-point meshes [7] of 4 × 4 × 4 for self-consistent calculations and 5 × 5 × 5 for DOS calculations. Spin-orbit coupling (SOC) was considered for all systems. The convergence criterion was set to 10^{-6} eV for band structure and DOS calculations.

Raman impurity model calculation:

In a given system of host crystal and impurity, the specific vibrational frequency of the with an atomic mass of M_i can be determined by its relative mass contrast (ε_i) and the specific vibrational frequency of both cations (M_1) and anions (M_2), where f is a force constant as a functional of mass-defect parameter can be determined as following equations[8]:

$$\varepsilon_i = 1 - \frac{M_i}{M_1} \tag{S1}$$

$$\omega_1{}^2 = \frac{2f}{M_1} \tag{S2}$$

$$\omega_2{}^2 = \frac{2f}{M_2} \tag{S3}$$

$$3\omega_1{}^2 + 3\omega_2{}^2 = 2\omega_{TO}{}^2 + 1\omega_{LO}{}^2 \tag{S4}$$

$$\omega_i{}^2 = \frac{2\omega_1{}^2 + (1-\varepsilon_i{}^2)\omega_2{}^2 + \left[4\varepsilon_i{}^2\omega_1{}^4 + (1-\varepsilon_1{}^2)^2\omega_2{}^4\right]^{\frac{1}{2}}}{2(1-\varepsilon_1{}^2)} \tag{S5}$$

Supplementary

Lattice thermal conductivity model:

Considering the mass and strain fluctuations as well as inherent lattice anharmonicity, the lattice thermal conductivity (κ_L) and the relaxation time (τ) are determined by the following equations: [9].

$$\kappa_L = \frac{1}{3}\int_0^{\omega_a} C_V(\omega) v_g^2 \tau d\omega \tag{S6}$$

$$\tau^{-1} = \tau_\varepsilon^{-1} + \tau_M^{-1} = \gamma^2\varepsilon^2 + \left(\frac{1}{\bar{M}}\right)^2 \Delta M^2 \tag{S7}$$

where C_V is the specific heat, ω_a is the cut-off frequency of acoustic phonons, v_g is the phonon group velocity, γ is the Grüneisen parameter measuring the strength of anharmonicity, $\bar{M}$ is the average atomic mass. ε and ΔM respectively represent the strain and mass fluctuations.

The total lattice strains (ε) and mass fluctuations (ΔM) can be determined by the following equations [10]:

$$\varepsilon^2 = A + B\varepsilon_{PD}^2 + C\varepsilon_{DS}^2 \tag{S8}$$

$$\Delta M^2 = D(\Delta M_{PD})^2 + E(\Delta M_{DS})^2 \tag{S9}$$

where A (for Umklapp scattering), B, C, D and E are the collection of physical constants, and subscripts PD for point defects and DS for dislocations.

Considering that acoustic phonons are the main contributors to κ_L, the model is based on acoustic modes[11] and on an average Grüneisen parameter (calculated from Leont'ev formula[12]), which leads the pre-factor A to be:

$$A = \frac{2}{(6\pi^2)^{\frac{1}{3}}} \frac{k_B \bar{V}^{\frac{1}{3}} \omega^2 T}{\bar{M} v_g v_p^2} \tag{S10}$$

where k_B is the Boltzmann constant, $\bar{V}$ is the average atomic volume, ω is the phonon frequency, T is the absolute temperature, $\bar{M}$ is the average atomic mass, v_p is the phase velocity, respectively. For simplicity, a Debye dispersion is used.

According to Klemens[13], the pre-factor B due to point defects can be written as:

$$B = \frac{\bar{V}\omega^4}{4\pi v_g v_p^2} 8(1+Q)^2 \tag{S11}$$

where Q=3.2 for an exclusion of the anharmonicity of the nearest elastic constants.

The strain field due to point defects can be determined by X-ray diffraction measurements (shift of diffraction peaks) [13]:

$$\varepsilon_{PD}^2 = \sum_i x_i \left(\frac{R_i - \bar{R}}{\bar{R}}\right)^2 = \sum_i x_i \left(\frac{11+r}{31-r}\right)^2 \left(\frac{a_i - \bar{a}}{\bar{a}}\right)^2 \tag{S12}$$

where x_i is the concentration of the solute i, R_i is the ionic radii of solute i, $\bar{R}$ is the average ionic radii, r is the Poisson ratio (calculated from Leont'ev formula[12]), a_i is the lattice constant of solute i and $\bar{a}$ is the average lattice constant, respectively.

The contribution to lattice strain due to 0D point defects and 1D dislocations are different[14]. Utilizing a Gaussian approximation, rocking curves enable an estimation on lattice strain fluctuations, and half width β_ε is given by [15]:

$$\beta_\varepsilon^2 = 8\left(\overline{\varepsilon_{DS}^2}\right) \ln 2 \, (\tan\theta)^2 \tag{S13}$$

where θ is the Bragg angle. The mean square strain ($\overline{\varepsilon_{DS}^2}$) along the direction $\bar{n}$ in the redial plane of the dislocation ($\bar{n}$ is the radial component of $\bar{N}$) is:

$$\overline{\varepsilon_{DS,n}^2} = \frac{1}{\pi l^2}\int_0^{2\pi}\int_{l_0}^{l} l\,[\varepsilon_{ll} + \varepsilon_{l\theta}\sin(\delta-\theta)\cos(\delta-\theta)]^2 dl d\theta = \frac{3-2r+3r^2+2(r^2-1)\cos(2\delta)}{32\pi^2(r^2-1)^2}\frac{b^2}{l^2}\ln\frac{l}{l_0} \tag{S14}$$

where the b is the Burgers vector, the l and l_0 are the upper and lower integration limits of the strain field, the $\varepsilon_{ll}=b\sin(\theta)/4\pi(1+r)l$ and $\varepsilon_{l\theta}=b\cos(\theta)/2\pi(1-r)l$. The l and l_0 are typically defined as $1/2N_D^{1/2}$ and one Burgers vector. Defining Δ as the angle between the dislocation glide plane normal and $\bar{N}$, and φ as the angle between $\bar{N}$ and $\bar{b}$, then $\cos^2(\delta)=\cos^2(\varphi)/(\cos^2(\varphi)+\cos^2(\Delta))$ where the angular relationships are shown in the literature[15]. The mean square strain along $\bar{N}$ is then $\overline{\varepsilon_{DS,N}^2}=\overline{\varepsilon_{DS,n}^2}(\cos^2(\varphi)+\cos^2(\Delta))$. The random orientation of dislocations is confirmed by our TEM observations, which rationalizes the following mathematical average as: both the integral average value of $\cos^2(\delta)$, $\cos^2(\varphi)$ and $\cos^2(\Delta)$ are 1/2. This leads $\cos(2\delta)$ to be zero.

This work utilizes Carruthers' Equation[16] to estimate the phonon relaxation time by edge dislocation scattering. This leads the pre-factor C to be:

$$C = \frac{2}{3}\frac{32\pi^2(r^2-1)^2}{3-2r+3r^2}\,\omega \ln\frac{\frac{1}{2N_D^{\frac{1}{2}}}}{b} \tag{S15}$$

where N_D is the dislocation density. The dislocation density can be solved when the lattice strain due to dislocation and the Burgers vector are known.

For the mass term due to point defects, the pre-factor D and ΔM_{PD} are[13]:

$$D = \frac{\bar{V}\omega^4}{4\pi v_g v_p^2} \tag{S16}$$

$$\Delta M_{PD}^2 = \sum_i x_i (M_i - \bar{M})^2 \tag{S17}$$

And the mass term for dislocations, the pre-factor E is [17]:

$$E = \frac{\bar{V}^{\frac{4}{3}}\omega^3}{v_g v_p} \tag{S18}$$

$$\Delta M_{DS}^2 = N_D(0 - \bar{M})^2 \tag{S19}$$

The parameters used for modeling are listed in Table S1.

Microstructures from X-ray diffraction peak broadening:

Various types of defects contribute to the broadening, intensity and shift of XRD diffraction peaks in different ways. Point defects usually distribute uniformly in the matrix, which leads to an overall expansion or contraction for releasing energy. This mainly results in a shift in Bragg diffraction positions (known as the Vegard's law). As for dislocations and interfaces, these lattice imperfections mainly induce strain fluctuations, leading to a broadening in diffraction peaks. The difference between relies on the factor that broadening due to dislocations increases with the diffraction order while that due to interfaces dose not. This enables the slope of peak broadening versus diffraction order to estimate the contribution of static lattice strains by dislocations, while the intercept corresponds to that by interfaces.

Supplementary

Table S1. Parameters used for the modeling.

Parameters	Description	Quantity	Ref.
a_i	Lattice parameters for solute i	a_{EuTe}=6.59 Å<br>a_{MnTe}=5.98 Å	18<br>18
$\bar{a}$	Average lattice parameters for alloy	Values in Fig. S1	This work
Npri	Number of atoms in primitive cell	2	-
$\bar{V}$	Average atomic volume of alloy	$\bar{a}^3/8$ m^3	-
$\bar{M}$	Average atomic mass of alloy	$M_{NayEuzMnxPb1\text{-}x\text{-}y\text{-}zTe}/(N\text{pri}\times 6.023\times 10^{23})$ kg	-
M_i	Average atomic mass of impurities	$M_{EuTe}/(N\text{pri}\times 6.023\times 10^{23})$ kg<br>$M_{MnTe}/(N\text{pri}\times 6.023\times 10^{23})$ kg	-
θ_D	Debye temperature	120 K	19
ω_a	Acoustic cut-off frequency	$(\theta_D/(N\text{pri})^{1/3})/(\hbar/k_B)$ Hz	-
γ	Gruneisen parameter	1.66	This work
x_i<br>z_i	Impurity concentration	$x_{Mn} \leq 0.03$<br>$z_{Eu} \leq 0.04$	This work
r	Poisson's ratio	0.281	This work
b	Burgers vector	$\bar{a}/2$	This work
N_D	Dislocation density	2.76×10^{-14} m^{-2} (annealing 1 h)<br>3.37×10^{-14} m^{-2} (annealing 4 h)<br>3.63×10^{-14} m^{-2} (annealing 16 h)<br>4.07×10^{-14} m^{-2} (annealing 48 h)<br>3.99×10^{-14} m^{-2} (annealing 72 h)<br>4.16×10^{-14} m^{-2} (annealing 120 h)<br>4.04×10^{-14} m^{-2} (annealing 360 h)<br>4.21×10^{-14} m^{-2} (annealing 720 h)<br>4.13×10^{-14} m^{-2} (annealing 1440 h)	This work

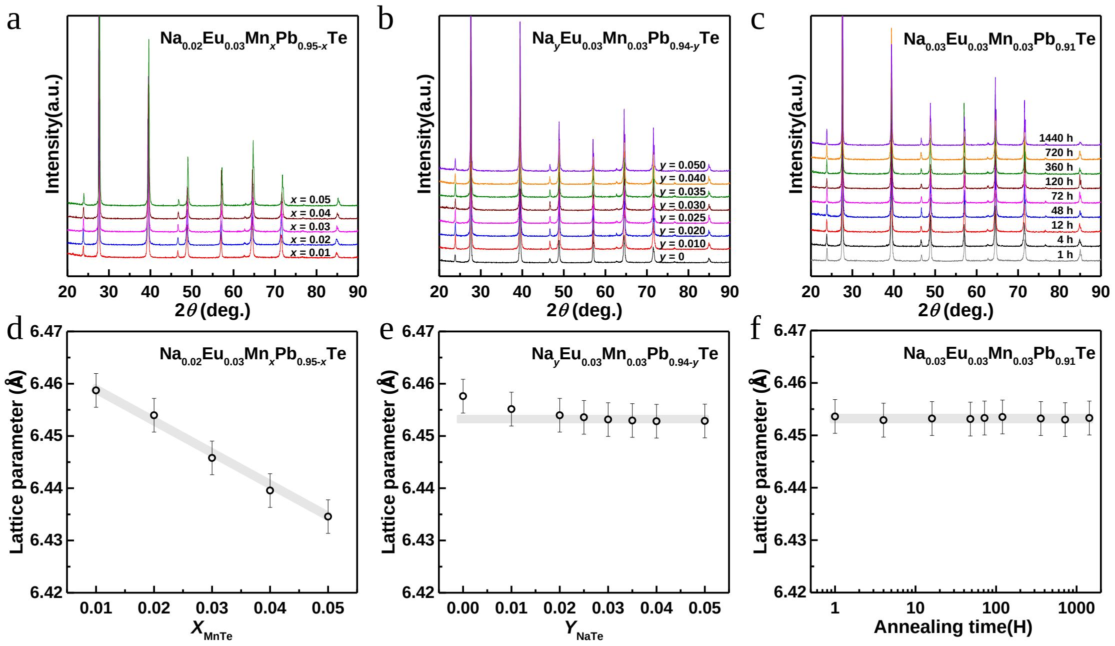

Fig. S1. XRD patterns (a-c) and lattice parameters (d-e) for $Na_{0.02}Eu_{0.03}Mn_xPb_{0.95-x}Te$ (a, d), $Na_yEu_{0.03}Mn_{0.03}Pb_{0.94-y}Te$ (b, e) and $Na_{0.03}Eu_{0.03}Mn_{0.03}Pb_{0.91}Te$ (c, f) with different annealing time.

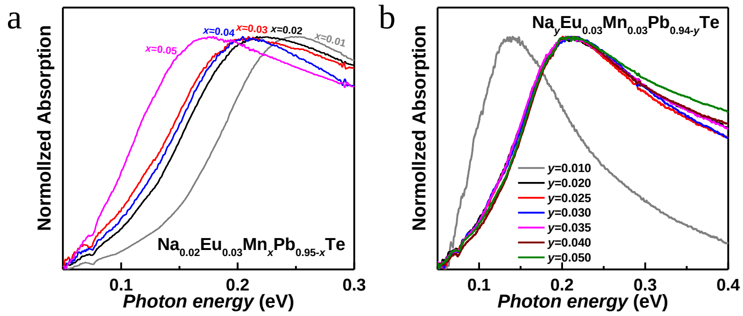

Fig. S2. Normalized optical absorption coefficient for $Na_{0.02}Eu_{0.03}Mn_xPb_{0.95-x}Te$ (a) and $Na_yEu_{0.03}Mn_{0.03}Pb_{0.94-y}Te$ (b) at room temperature.

Supplementary

Table S2. Room temperature Hall carrier concentration (n_H), inertial effective mass (m_I*), plasma energy and density (g/cm^3) for $Na_{0.02}Eu_{0.03}Mn_xPb_{0.95-x}Te$ and $Na_yEu_{0.03}Mn_{0.03}Pb_{0.94-y}Te$.

Matierials	n_H (10^{20}cm^{-3})	m_I* (m_e)	Plasma energy (eV)	Density (g/cm^3)
$Na_{0.02}Eu_{0.03}Mn_{0.01}Pb_{0.94}Te$	1.07	0.11	0.25	8.11
$Na_{0.02}Eu_{0.03}Mn_{0.02}Pb_{0.93}Te$	1.06	0.14	0.22	8.13
$Na_{0.02}Eu_{0.03}Mn_{0.03}Pb_{0.92}Te$	1.04	0.17	0.21	8.15
$Na_{0.02}Eu_{0.03}Mn_{0.04}Pb_{0.91}Te$	1.05	0.19	0.20	8.15
$Na_{0.02}Eu_{0.03}Mn_{0.05}Pb_{0.90}Te$	1.01	0.22	0.18	8.15
$Na_{0.01}Eu_{0.03}Mn_{0.03}Pb_{0.93}Te$	0.52	0.17	0.14	7.97
$Na_{0.025}Eu_{0.03}Mn_{0.03}Pb_{0.915}Te$	1.04	0.17	0.20	7.98
$Na_{0.03}Eu_{0.03}Mn_{0.03}Pb_{0.91}Te$	1.06	0.16	0.21	8.01
$Na_{0.035}Eu_{0.03}Mn_{0.03}Pb_{0.905}Te$	1.06	0.16	0.21	8.02
$Na_{0.04}Eu_{0.03}Mn_{0.03}Pb_{0.90}Te$	1.05	0.16	0.21	8.03
$Na_{0.05}Eu_{0.03}Mn_{0.03}Pb_{0.89}Te$	1.05	0.15	0.21	7.97

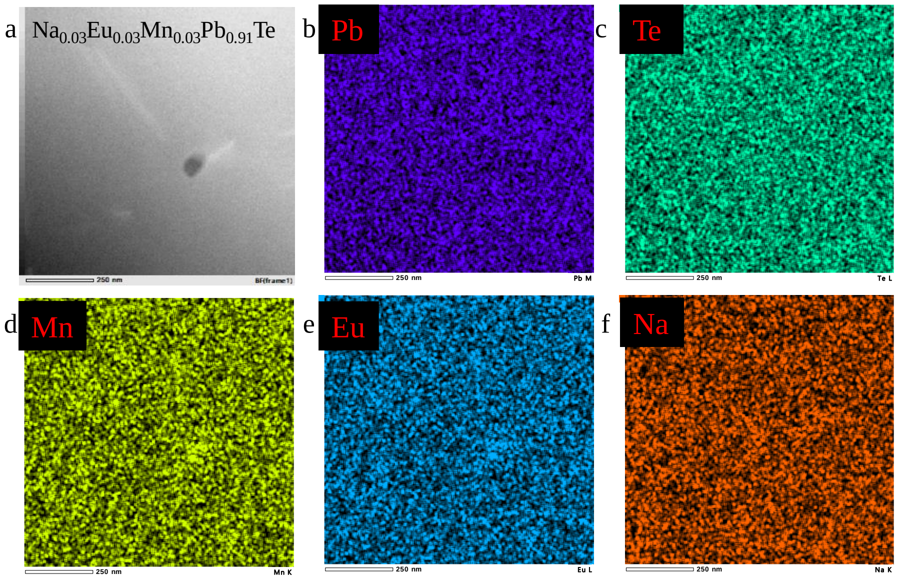

Fig. S3. TEM images and EDS composition mappings for $Na_{0.03}Eu_{0.03}Mn_{0.03}Pb_{0.91}Te$.

Supplementary

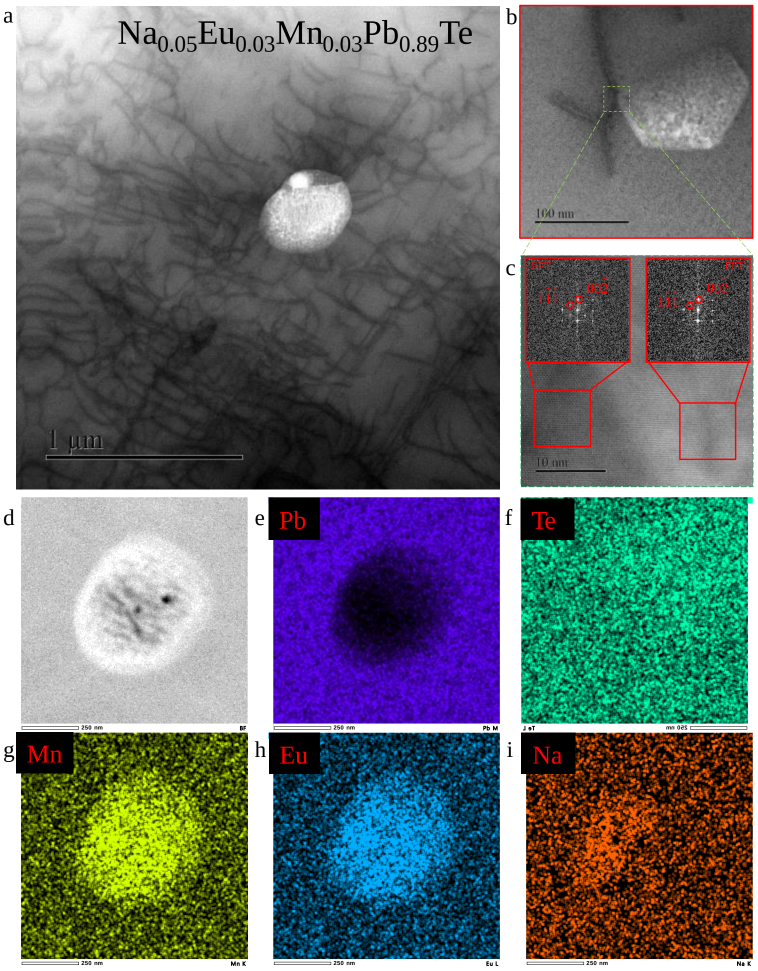

Fig. S4. Low-magnification STEM images (a) and high-magnification STEM image(b), electron diffraction patterns(c) and EDS composition mappings(d-i) for $Na_{0.05}Eu_{0.03}Mn_{0.03}Pb_{0.89}Te$, indicating the coexistence of both dense dislocations and nano-precipitates.

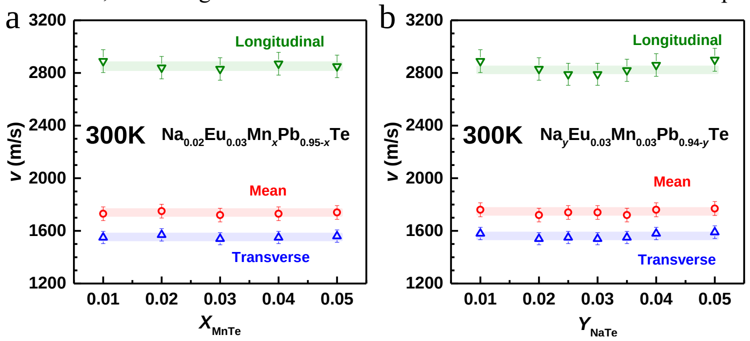

Fig. S5. MnTe (a) and NaTe (b) contents dependent sound velocity for $Na_{0.02}Eu_{0.03}Mn_xPb_{0.95-x}Te$ and $Na_yEu_{0.03}Mn_{0.03}Pb_{0.94-y}Te$ at room temperature.

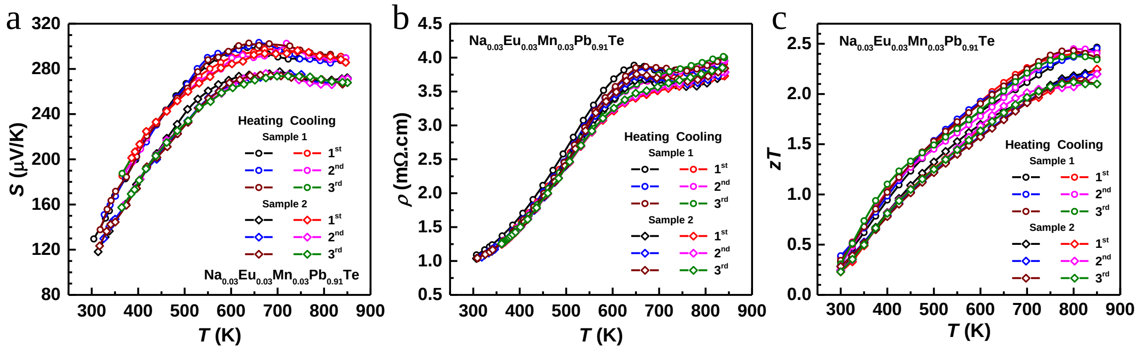

Fig. S6. Repeated measurements for the high-zT composition $Na_{0.03}Eu_{0.03}Mn_{0.03}Pb_{0.91}Te$ showing a highly reproducible and stable thermoelectric properties.

Supplementary

Supplementary References:

1. Pei, Y.; LaLonde, A. D.; Wang, H.; Snyder, G. J. *Energy Environ. Sci.* **2012,** 5, (7), 7963.
2. Blachnik, R.; Igel, R. *Z Naturforsch B* **1974,** 29, (9-10), 625-629.
3. Kresse, G.; Hafner, J. *Phys. Rev. B.* **1993,** 47, (1), 558-561.
4. Kresse, G.; Furthmüller, J. *Phys. Rev. B.* **1996,** 54, (16), 11169.
5. Blöchl, P. E. *Phys. Rev. B.* **1994,** 50, (24), 17953-17979.
6. Oganov, A. R.; Glass, C. W. *J Chem Phys* **2006,** 124, (24), 244704.
7. Monkhorst, H. J.; Pack, J. D. *Phys. Rev. B.* **1976,** 13, (12), 5188-5192.
8. Lucovsky, G.; Brodsky, M. H.; Burstein, E. *Phys. Rev. B.* **1970,** 2, (8), 3295-3302.
9. Klemens, P. G. **1958,** 7, 1-98.
10. Wu, Y.; Chen, Z.; Nan, P.; Xiong, F.; Lin, S.; Zhang, X.; Chen, Y.; Chen, L.; Ge, B.; Pei, Y. *Joule* **2019,** 3, (5), 1276-1288.
11. Toberer, E. S.; Zevalkink, A.; Snyder, G. J. *J. Mater. Chem.* **2011,** 21, (40), 15843.
12. KEONTEV, K. *Sov Phys Acoust+* **1981,** 27, (4), 309-313.
13. Klemens, P. G. *Proceedings of the Physical Society* **1955,** A68, (12), 1113-1128.
14. Ungár, T. *Scripta Mater* **2004,** 51, (8), 777-781.
15. Hordon, M.; Averbach, B. *Acta Metall Mater* **1961,** 9, (3), 237-246.
16. Carruthers, P. *Phys. Rev.* **1959,** 114, (4), 995-1001.
17. Klemens, P. G. *Solid State Phys.* **1958,** 7, 1-98.
18. P., V., PAULING FILE in Inorganic Solid Phases. SpringerMaterials: 2016.
19. Morelli, D. T.; Jovovic, V.; Heremans, J. P. *Phys Rev Lett* **2008,** 101, (3), 035901.
